# Work up of fatty liver by primary care physicians, review

**DOI:** 10.1016/j.amsu.2020.01.001

**Published:** 2020-01-11

**Authors:** Rishi Rikhi, Tavankit Singh, Jamak Modaresi Esfeh

**Affiliations:** a Cleveland Clinic, Department of Internal Medicine, 9500 Euclid Avenue, Cleveland, OH, 44195, USA; b Cleveland Clinic, Department of Gastroenterology, Hepatology & Nutrition, 9500 Euclid Avenue, Cleveland, OH, 44195, USA

**Keywords:** NAFLD, Primary health care

## Abstract

Nonalcoholic fatty liver disease (NAFLD) is an overarching term that refers to abnormal deposition of lipids in the liver and is used to describe the spectrum of disease ranging from hepatic steatosis to nonalcoholic steatohepatitis to cirrhosis. NAFLD is the most common cause of chronic liver disease and the second most common cause of cirrhosis. Although the pathophysiology is not completely understood, there is a strong link between NAFLD and metabolic syndrome. This review focuses on the workup of NAFLD in the primary care setting, from differential diagnoses to assessing fibrosis via predictive models that use commonly used laboratory values, biomarkers, and imaging. The purpose of this review article is to provide a set of screening and diagnostic tools for all primary care physicians in order to better manage patients with NAFLD.

## Highlights

• Nonalcoholic fatty liver disease is the most common cause of chronic liver disease.
• Primary care providers have an important role in diagnosis.
• Prediction models and imaging have helped estimate fibrosis.
• Main treatment involves lifestyle modifications and managing comorbid conditions.

## Abbreviations

NAFLD: Nonalcoholic fatty liver disease
US: United States
CLD: Chronic liver disease
HDL: High-density lipoprotein cholesterol
PCPs: Primary care providers
NASH: Nonalcoholic steatohepatitis
SS: Simple steatosis
VLDL: Very low-density lipoprotein
JNK: Jun N terminal kinase
PNPLA3: Patatin-like phospholipase 3
TM6SF2: Transmembrane 6 superfamily member 2
AST: Aspartate aminotransferase
ALT: Alanine transaminase
HU: Hounsfield units
HELLP: Hemolysis, elevated liver enzymes, low platelet count
AASLD: The American Association for Study of Liver Disease
FIB-4: Fibrosis-4 index
VCTE: Vibration controlled transient elastography
NFS: NAFLD fibrosis score
BMI: Body mass index
ELF: Enhanced Liver Fibrosis
TIMP-1: Metalloproteinases
PIIINP: Propeptide of procollagen type III
VCTE: Vibration-controlled transient elastography
MRE: Magnetic resonance elastography
MRI: Magnetic resonance imaging
AGA: American Gastroenterology Association

## 1 Introduction

Chronic liver disease (CLD) is the 12th leading cause of death in the United States (US) [1]. The most common cause of CLD not just in the US, but worldwide is nonalcoholic fatty liver disease (NAFLD) [[2], [3], [4]]. NAFLD is currently the second most common etiology of cirrhosis in patients undergoing liver transplantation [5] and is projected to become the leading cause of liver transplant by 2020 [6,7]. The increase in prevalence of NAFLD, examined by the National Health and Nutrition Examination Surveys from 1988 to 2008 illustrating that NAFLD as a cause of CLD rose from 46.8% in 1988 to 75.1% in 2008 parallels the rise in prevalence of obesity, diabetes mellitus and hypertension during the same time period [4]. In addition to hypertension, diabetes and obesity; hypertriglyceridemia and low levels of high-density lipoprotein cholesterol (HDL) have also been found to be risk factors for the development of NAFLD [8].

Several of the above risk factors for NAFLD are chronic conditions managed by primary care providers (PCPs) [9]. Thus, PCPs usually are the ones who have the opportunity to diagnose patients with NAFLD and manage it initially [9]. Yet, surveys have found that 33% of PCPs underestimated the prevalence of NAFLD [9], 69% did not identify NAFLD as a clinically important condition and 53% were uncomfortable with the management of NAFLD [9]. Hence, there is an urgent need to educate PCPs on the epidemiology and work up of this very common disease in order to provide more effective care for patients with NAFLD. This review article provides an overview of NAFLD and the recommended workup in the primary care setting.

## 2 What is nonalcoholic fatty liver disease?

NAFLD is an overarching term that refers to abnormal deposition of lipids in the liver and is used to describe the spectrum of disease ranging from hepatic steatosis to nonalcoholic steatohepatitis (NASH) to cirrhosis [11]. By definition, a diagnosis of NAFLD cannot be made in an individual with other etiologies of fatty liver disease (discussed below) or in patients with excessive alcohol intake, described as 20 g and 10 g a day for men and women, respectively [11]. For reference, 12 ounces of beer, 5 ounces of wine, and 1.5 ounces of 80 proof liquor are all equal to 14 g of alcohol [12]. Simple steatosis (SS) refers to excessive lipid deposition in >5% hepatocytes without hepatocellular injury [11,13]. While the course of SS is relatively benign [14], approximately 20% of individuals with SS will progress to the more aggressive variant of NAFLD-called NASH, which includes the presence of steatosis, lobular inflammation and hepatocellular injury [15]. Hepatocellular injury is characterized by hepatocyte ballooning, a term used to describe hepatocytes that have lost their sharp angles and have a non-vacuolar cytoplasm [16]. Progressive inflammation leads to activation of stellate cells in the liver which deposit collagen in the hepatic lobules. This process is known as fibrosis and fibrosis is staged 0–4 depending on the extent and distribution (Table 1) [17]. Approximately 20% of patients with NASH and 38% patient with NASH and fibrosis develop cirrhosis [18]. Unfortunately, to date, we do not have any tool to predict which patients with NAFLD will progress to cirrhosis.

**Table 1:** NASH stages of fibrosis.

Fibrosis Stage	
F 0	No fibrosis
F 1	Perisinusoidal or periportal fibrosis
F 2	Perisinusoidal and portal/periportal fibrosis
F 3	Bridging fibrosis
F 4	Cirrhosis

## 3 How does NAFLD develop?

**Fig. 1:**
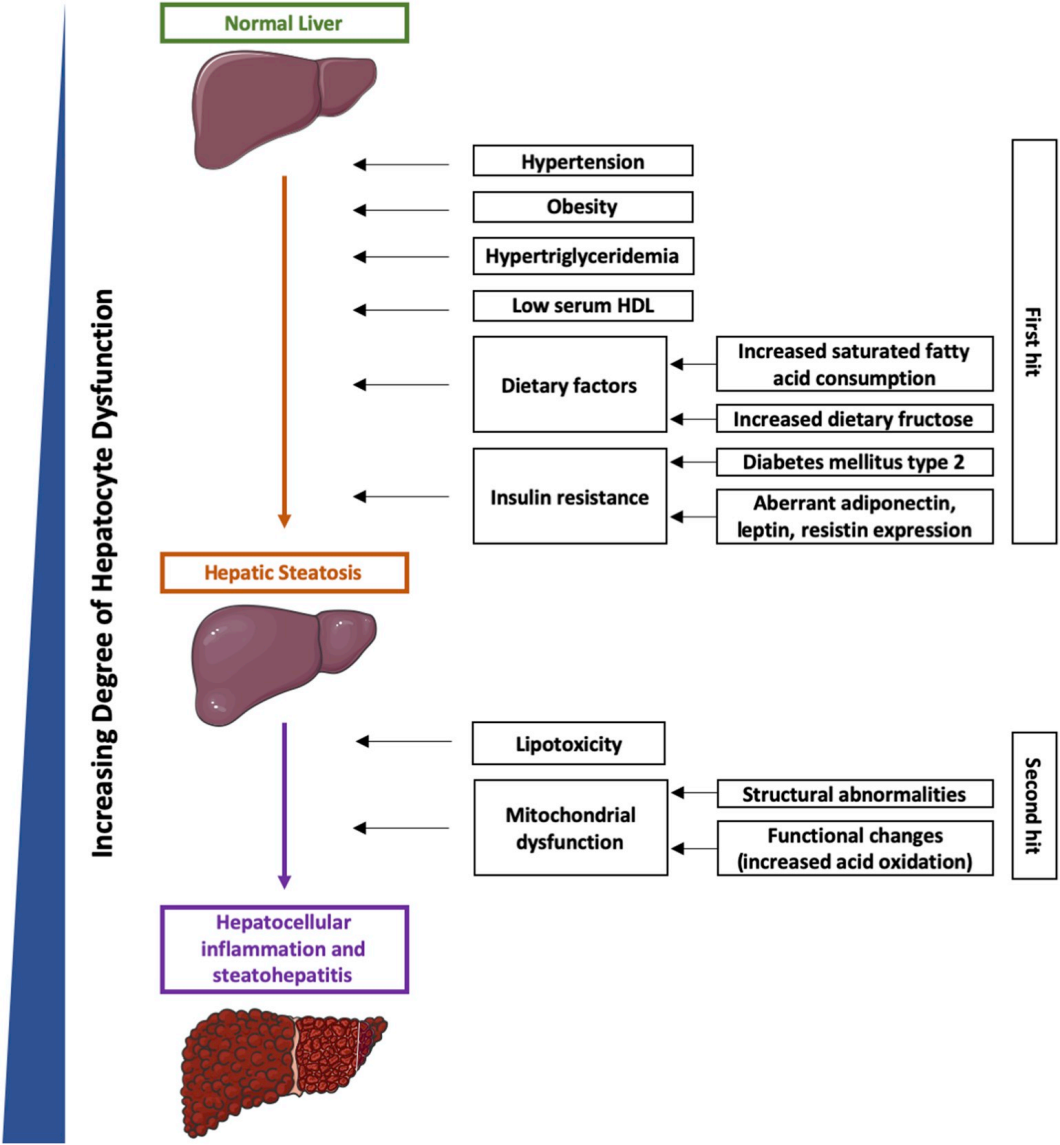
Pathogenesis of NAFLD using 2 hit hypothesis.

The pathophysiology of NAFLD is complex and not fully understood. One of the leading theories is the “two-hit” hypothesis (Fig. 1). Here, the first hit leads to hepatic steatosis and the second hit results in steatohepatitis and hepatocellular injury. The liver allows for lipid homeostasis and this balance can be offset in obesity or in individuals with a dietary intake high in saturated fatty acids and fructose, leading to increased fatty acid deposition in the liver, resulting in hepatic steatosis. Studies have found abdominal obesity (measured by waist circumference) to be more strongly associated with NAFLD, as visceral fat has higher rates of lipolysis, leading to increased delivery of fatty acids to the liver [19]. Another factor that leads to increased uptake of free fatty acids and triglycerides in the liver is insulin resistance [13]. Insulin normally suppresses hepatic production of very low-density lipoprotein (VLDL), which is rich in triglycerides; thus, insulin resistance leads to hypertriglyceridemia [19]. Additionally, the higher amount of VLDL in the bloodstream leads to decreased HDL [19]. Hormones, such as adiponectin, leptin, and resistin regulate insulin activity and aberrant expression of these hormones further leads to the development of NAFLD [20,21].

Hepatic inflammation is thought to occur from lipotoxicity and mitochondrial dysfunction [22]. Mitochondrial dysfunction includes structural and functional changes, which impairs fat homeostasis leading to increased inflammation and lipid-derived toxic metabolites [22]. Lipotoxicity occurs from saturated fatty acids that activate the Jun N terminal kinase (JNK) pathway, resulting in hepatocyte death [22]. The excess fatty acids from lipolysis leads to increased acid oxidation, resulting in mitochondrial dysfunction [22]. The resulting inflammation leads to activation of Kupffer cells, which release cytokines that further damage hepatocytes [22]. The inflammatory process converts hepatic stellate cells to myofibroblasts, resulting in hepatic fibrosis [22].

## 4 Risk factors for NAFLD

There is a strong association between NAFLD and metabolic syndrome [19,23]. This syndrome is defined as having three of the following conditions: diabetes mellitus, low HDL, hypertriglyceridemia, hypertension, and increased abdominal waist circumference [19]. The exact definitions for each of these conditions varies based on the organization or society [24]. The International Diabetes Federation guidelines from 2005 are commonly used in practice (Table 2) [24].

**Table 2:** The international diabetes federation guidelines 2005.

Waist Circumference	>80 cm in women and <90 cm in men
Lipid Dysregulation	Triglycerides >150, HDL-C < 40 in men and <50 in women
Hypertension	Systolic>130 mm Hg, Diastolic >85, or patient on hypertension medications
Hyperglycemia	Glucose over 100 mg/dL or diabetic

Patients with increased waist circumference, fasting glucose, blood pressure, and triglycerides have a 4.9-fold, 2.1-fold, 1.8-fold, and 1.6-fold greater risk of NAFLD, respectively [19]. The prevalence of NAFLD varies from 45% to 75% in diabetics and over 50% in patients with hypertension [23]. The prevalence of NAFLD in obese patients is 80–90% and approximately 90% in patients with hyperlipidemia [25].

While most patients with NAFLD have metabolic syndrome, seminal research has focused on a population of patients who are not obese but have NAFLD [26]. Often, these cases, commonly referred to as “lean NAFLD” are overlooked as they do not fit the typical NAFLD presentation [26]. Lean NAFLD illustrates the complexity of NAFLD pathophysiology and underscores the interplay between genetics and metabolic syndrome in the development of NAFLD [26]. While limited information exists on the why patients with normal body weights develop NAFLD, research has shown that lean NAFLD is more prevalent among the Asian population [26]. Additionally, patients with lean NAFLD still have higher amounts of abdominal fatty tissue, although their overall body weight is normal [26].

There is also a strong genetic component associated with the development of NAFLD with Latin Americans carrying the highest burden of NAFLD, and African Americans the lowest [27,28]. A prospective study of 320 individuals in the outpatient setting found the prevalence of NAFLD to be 58.3% in Hispanics, 44.4% in Caucasians, and 35.1% in African Americans [29].

To further understand the genetic risk associated with NAFLD, research on polymorphisms in regulatory proteins involved in hepatic lipid metabolism and insulin signaling is currently underway [13]. Patatin-like phospholipase 3 (PNPLA3) and transmembrane 6 superfamily member 2 (TM6SF2) are two well characterized genes involved in the pathogenesis of NAFLD [22]. PNPLA3 encodes adiponutrin, a protein that aids in triglyceride metabolism and TM6SF2 encodes TM6SF2 protein that aids in secretion of VLDL from the liver [22]. Polymorphisms of PNPLA3 and TM6SF2 are associated with increased hepatic triglyceride accumulation and hepatic steatosis [22].

In addition to risk factors mentioned above, there are also uncommon causes of NAFLD [30]. There are several disorders of lipid metabolism that lead to NAFLD: abetalipoproteinemia, familial hypobetalipoproteinemia, familial combined hyperlipidemia, glycogen storage disease, Weber-Christian disease, and congenital lipodystrophy [30]. Certain nutritional causes, including total parenteral nutrition, surgical weight loss, and starvation can lead to NAFLD as well [30]. Long term total parenteral nutrition results in a depletion of carnitine and choline, key players in fatty acid transport and lipid storage, leading to steatosis [30]. Surgical weight loss leads to an increase in free fatty acids and starvation results in protein depletion, including apolipoprotein synthesis, both leading to NAFLD [30]. Lastly, several medications have been shown to promote NAFLD, including amiodarone, tamoxifen, methotrexate, corticosteroids, and highly active antiretroviral therapy [30].

## 5 Clinical presentation and diagnostic modalities

Patients with NAFLD may present to their PCP with complaints of fatigue and right upper quadrant pain; however, many will not have any symptoms, although the majority will be overweight [31]. Evaluation of liver enzymes is helpful, as NAFLD is the most common cause of chronically elevated liver enzymes [32]. Aspartate aminotransferase (AST), found in hepatocellular mitochondria, and alanine transaminase (ALT), found in hepatocellular cytosol, are released during times of liver injury [33]. Patients with NAFLD typically have an ALT level that is higher than AST except in patients with advanced fibrosis where AST might be equal to or higher than ALT. The degree of elevation of AST and ALT does not correlate with the quantity of hepatic fat deposition or severity of fibrosis. Despite having NAFLD, some patients can have normal AST/ALT levels. In fact, patients with normal ALT values can exhibit the full spectrum of NAFLD [31]. Neither AST nor ALT are a reflection of synthetic function of the liver. As a matter of fact, “liver function tests” is a misnomer, as liver enzymes are not a reflection of liver function, but rather, hepatocyte integrity [34]. The blood work that can show synthetic function of the liver include albumin, INR, and bilirubin [34]. These laboratory values are a measure of synthetic liver function and can be normal in patients with NAFLD [11].

**Fig. 2:**
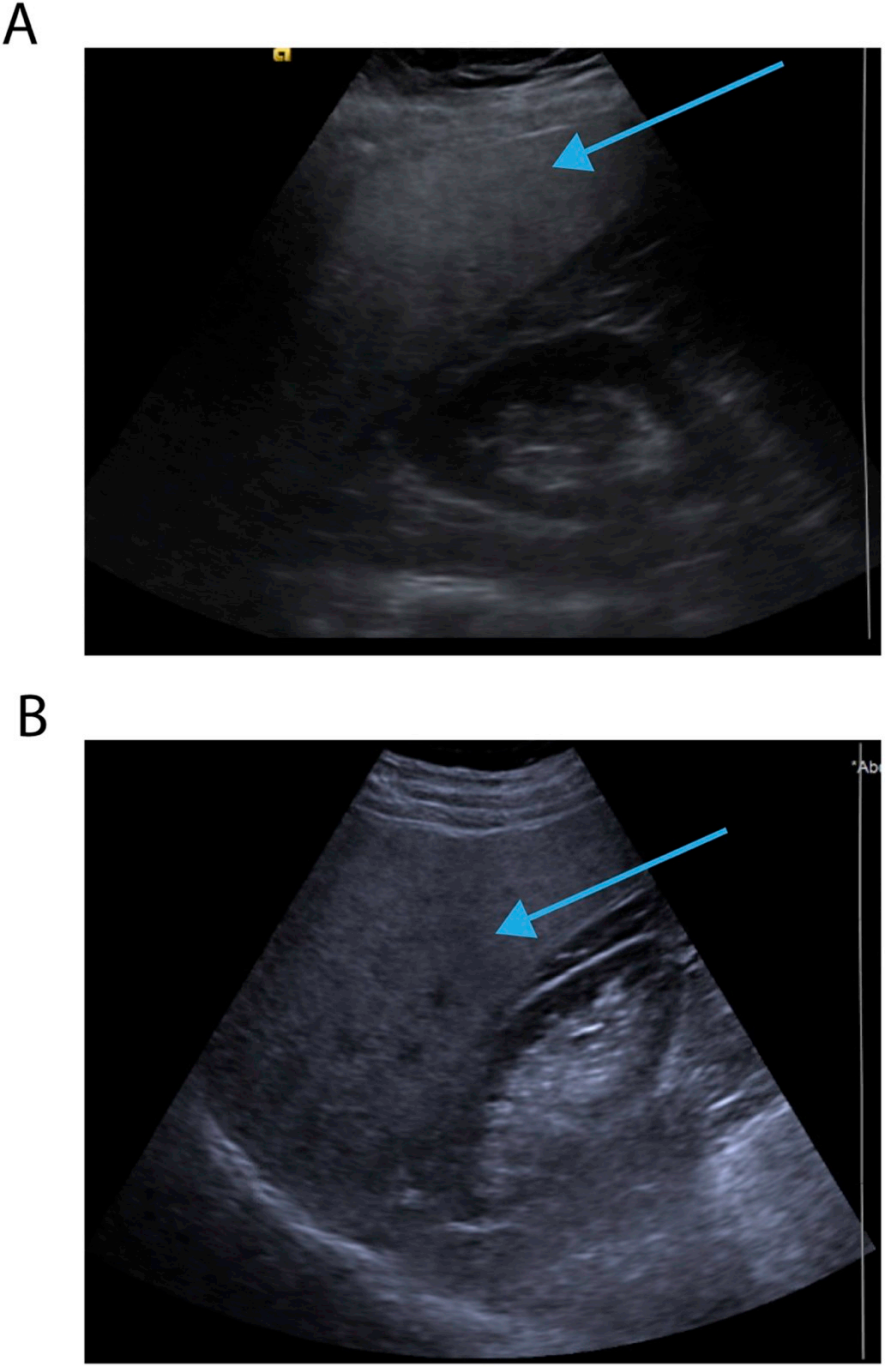
Ultrasonographic evidence of hepatic steatosis. The blue arrow highlights the increased echogenicity of the liver. (For interpretation of the references to colour in this figure legend, the reader is referred to the Web version of this article.)

Patients who have an incidental finding of hepatic steatosis on imaging should be evaluated for NAFLD [11]. While screening for NAFLD in high-risk groups is not recommended, right upper quadrant ultrasound or abdominal CT images may be ordered in patients for other reasons; and subsequently, NAFLD may be incidentally diagnosed [11]. Approximately 11% of individuals who have thoracic or abdominal imaging for non-liver related reasons would have incidental findings of hepatic steatosis [11]. Ultrasound is a noninvasive imaging modality used to view the echogenicity of the liver parenchyma (Fig. 2) [35]. The deposition of fat in the liver increases the echogenicity (Fig. 2) [35]. Normally, the echogenicity of the liver, spleen, and renal cortex are similar [36]. Therefore, using the spleen and renal cortex as a comparison, increased echogenicity of the liver can be assessed with ultrasound (Fig. 2) [36]. CT imaging uses Hounsfield units (HU) to measure attenuation of organs and vasculature [36]. The liver normally has increased attenuation compared to the spleen and intrahepatic vasculature [36]. Fatty liver causes decreased attenuation; thus, if the liver is 10 HU less than the spleen or if the liver has a total attenuation less than 40 HU, fatty liver is suspected [36]. Ultrasound and non-contrast enhanced CT imaging are useful modalities for detecting fatty liver in patients with moderate to severe cases of hepatic steatosis; however, they are not effective at detecting milder cases [36]. In fact, once fatty liver has been detected by US imaging, more than 20% of the liver is fat content [37].

## 6 Differentials

In order for a diagnosis of NAFLD to be made, other causes of liver disease must be excluded first, including viral hepatitis, autoimmune hepatitis, Wilson disease, hemochromatosis, alpha-1 antitrypsin deficiency, and alcoholic liver disease [11]. The patient's clinical picture of diabetes mellitus, obesity, and dyslipidemia can help to narrow the differential diagnoses [11]. Also, alcoholic liver disease must be ruled out by assessing the patient's history of alcohol use [11]. If NAFLD is still likely, other causes of hepatic steatosis must be investigated (Table 3) [11]. Patients on parental nutrition or malnourished may develop macrovesicular steatosis [11]. Wilson disease, hepatitis C infection, lipodystrophy, and abetalipoproteinemia can all cause macrovesicular steatosis [11]. Patients who are pregnant may develop acute fatty liver of pregnancy or HELLP (hemolysis, elevated liver enzymes, low platelet count) syndrome, leading to microvesicular steatosis [11]. Additionally, Reye's syndrome and genetic metabolic diseases can lead to microvesicular steatosis [11]. Iron studies can be useful in differentiating hemochromatosis, but serum ferritin may be elevated in NAFLD patients as well [11]. When elevated in NAFLD patients, the ferritin and transferrin saturation are only mildly elevated. Approximately 21% of NAFLD patients will have high antibody titers (anti-nuclear antibodies > 1:160 and anti-smooth muscle antibodies > 1:40); yet, positive antibodies and higher titer values are not associated with advanced disease progression [11]. Specific lab tests should be ordered by PCPs based on the clinical presentation of the patient, family history, and patient's pretest probability [38].

**Table 3:** Differentials of NAFLD.

Condition	Clinical History
Alcoholic liver disease	Alcohol consumption: • >20 g a day for men • >10 g a day for women
Medication induced fatty liver disease	Elevation of AST and ALT will coincide with medication use. • Common medications include: • Lipid lowering agents (mipomersen and lomitapide) • Antiarrhythmics (amiodarone) Immunosuppressive agents (methotrexate, tamoxifen, and corticosteroids) • Antiepileptics (sodium valproate) • Antiretrovirals
Starvation	• Clinical history of BMI<18.5 • Unintentional weight loss • Poor oral intake
Parenteral nutrition	• Current use of parenteral nutrition • History of recent use of parenteral nutrition
Hepatitis C	• History of intravenous drug use • History of risky sexual practices • Lab tests supporting Hepatitis C infection
Acute fatty liver of pregnancy	• 3rd trimester pregnancy or early postpartum • Right upper quadrant pain • Jaundice • Febrile • Nausea and anorexia
HELLP syndrome	• 3rd trimester pregnancy • Headaches and visual disturbances • Nausea and vomiting • Abdominal pain
Reye's Syndrome	• Children • Use of aspirin • Recent viral infection • Seizures
Abetalipoproteinemia	• Diagnosed early in life • Failure to thrive • Neurological symptoms • Acanthocytosis • Foul smelling stools
Wilson Disease	• Younger than 55 years of age • Psychiatric symptoms • Kayser-Fleischer rings
Hemochromatosis	• Skin pigmentation • Diabetes • Cardiomegaly

## 7 Screening

The data regarding screening individuals with risk factors for NAFLD is conflicting [11]. There is a lack of information on the effectiveness of screening tests, such as ultrasound imaging or biochemical studies, as well as diagnostic tests and treatment. Recent studies have suggested that routine screening in high risk groups, such as patients with diabetes mellitus or a family history of NASH, is not cost effective and should not be done in the primary care setting [39,40]. The American Association for Study of Liver Disease (AASLD) guidelines currently do not recommend routine screening for NAFLD, even in patients with risk factors and also do not recommend screening family members of NAFLD patients [11].

• *Routine Screening for NAFLD in high-risk groups attending primary care, diabetes, or obesity clinics is not advised at this time because of uncertainties surrounding diagnostic tests and treatment options, along with lack of knowledge related to long-term benefits and cost-effectiveness of screening* [11]
• *There should be a high index of suspicion for NAFLD and NASH in patients with type 2 diabetes. Clinical decision aids such as NAFLD fibrosis score or fibrosis-4 index (FIB-4) or vibration controlled transient elastography (VCTE) can be used to identify those at low or high risk for advanced fibrosis (bridging fibrosis or cirrhosis)* [11]

## 8 Management of NAFLD

Given that the degree of fibrosis is linked to long term outcomes and mortality in NAFLD patients [41], one of the first steps to do after diagnosing a patient with NAFLD is to assess the stage of fibrosis. While liver biopsy remains the gold standard test for establishing the stage of fibrosis, in order to avoid an invasive procedure that carries a risk of pain and bleeding, a number of prediction models that use demographic variables and laboratory values have been developed (Table 4, Table 5, Table 6). In addition, over the last few years, imaging techniques like transient elastography and magnetic resonance elastography have been developed to replace or serve as an adjunct to the prediction models to estimate the fibrosis stage.

**Table 4:** Non-invasive blood tests—serum calculators.

NFS	Score	Meaning	Sensitivity	Specificity	PPV	NPV
	<-1.455	Absence of significant fibrosis	82%	77%	56%	93%
	>0.675	Presence of significant fibrosis	51%	98%	90%	85%
FIB-4						
	<1.3	Absence of significant fibrosis	74%	71%	43%	90%
	>2.67	Presence of advanced fibrosis	33%	98%	80%	83%

**Table 5:** Non-invasive blood tests—serum biomarker tests.

FibroSURE	Score	Meaning	Sensitivity	Specificity	PPV	NPV
	>0.3	Detection of bridging fibrosis or cirrhosis	92%	71%	33%	98%
	>0.7	Detection of bridging fibrosis or cirrhosis	25%	97%	60%	89%
Hepascore						
	>0.5–0.55	Detection of significant fibrosis	70%	79%	78%	71%
ELF						
	>9.8	Severe fibrosis	86.7%	92.5%	72%	97%

**Table 6:** Assessment of fibrosis.

*Non-Invasive Blood Tests*	
NAFLD fibrosis score (NFS)	Age, body mass index (BMI), presence of diabetes, AST, ALT, platelets, and albumin
Fib-4 index	Platelet count, patient's age, AST, and ALT
FibroSURE	ALT, α2‐macroglobulin, apolipoprotein A1, γ‐glutamyl transferase, haptoglobin, total bilirubin, age, and gender
Hepascore	α2‐macroglobulin, hyaluronic acid, γ‐glutamyl transpeptidase, total bilirubin, age, and gender
Enhanced Liver Fibrosis (ELF)	Hyaluronic acid, tissue inhibitors of metalloproteinases (TIMP-1), and amino-terminal propeptide of procollagen type III (PIIINP)
*Non-Invasive Imaging*	
Vibration-controlled transient elastography (VCTE)	Ultrasonic waves to detect liver stiffness
Magnetic resonance elastography (MRE)	Magnetic resonance imaging (MRI) with mechanical waves to measure liver stiffness
*Invasive*	
Liver Biopsy	Percutaneous liver biopsy and transvenous liver biopsy

## 9 Prediction models based on demographic variables and/or laboratory values

The prediction models typically differentiate between presence of advanced fibrosis (stage 3–4 fibrosis) and absence of advanced fibrosis (i.e. presence of stage 0–2 fibrosis) [42]. The NAFLD fibrosis score (NFS) uses age, body mass index (BMI), presence of diabetes, AST, ALT, platelets, and albumin [43]. This model uses a score of less than −1.455 (negative predictive value 88% in validation group) to represent stages F0–F2 and a score of greater than 0.675 (positive predictive value of 82% in validation group) to represent stages F3–F4 (Table 4, Table 6) [43]. A recent study evaluating the cost-effectiveness of fibrosis risk stratification tools found that NFS was the most cost effective in the primary care setting [44]. NFS is 90% accurate in detecting the absence or presence of fibrosis [43].

Another tool for hepatic fibrosis assessment is the FIB-4 index that uses platelet count, patient's age, AST, and ALT to predict fibrosis [45]. The FIB-4 score was originally used to estimate fibrosis stage in patients with hepatitis C virus infection but was subsequently validated for NAFLD patients too [46]. FIB-4 values less than 1.6 have a 93.2% negative predictive value and values greater than 3.6 have a 90.8% positive predictive value for detecting cirrhosis [47]. Both NFS and Fib-4 have been validated by several studies, including McPherson et al., and can be used in NAFLD patients to predict hepatic fibrosis (Table 4, Table 6) [48].

FibroSURE, Hepascore, and Enhanced Liver Fibrosis (ELF) score are three serum studies that measure direct biomarkers of fibrosis to predict hepatic fibrosis in NAFLD (Table 5, Table 6) [49]. FibroSURE uses ALT, α2‐macroglobulin, apolipoprotein A1, γ‐glutamyl transferase, haptoglobin, total bilirubin, age, and gender [49]. Hepascore uses α2‐macroglobulin, hyaluronic acid, γ‐glutamyl transpeptidase, total bilirubin, age, and gender [50]. The ELF scoring system uses hyaluronic acid, tissue inhibitors of metalloproteinases (TIMP-1), and amino-terminal propeptide of procollagen type III (PIIINP) in order to predict fibrosis [51]. These tests are expensive as they include special laboratory testing, which is not routinely done when evaluating patients with CLD whereas the NAFLD fibrosis score and FIB-4 score use the laboratory values that are routinely done in CLD patients.

### 9.1 Non-invasive imaging

Measurement of liver stiffness via vibration-controlled transient elastography (VCTE) is a useful noninvasive ultrasound-based tool to assess for liver fibrosis in patients with NAFLD (Table 6) [52]. VCTE is not capable of diagnosing NASH, but rather, can be used to estimate the degree of fibrosis [53]. VCTE works by using ultrasonic waves to detect liver stiffness, which has a high correlation with hepatic fibrosis [52]. Using VCTE, a cut of value of 10.3 kPa has a 99% negative predictive value and 46% positive predictive value for cirrhosis [53]. This modality previously had limitations as the probe was unable to measure adequate depths [52]. In patients with a BMI greater than 30 kg/m^2^ have a VCTE failure rate of 22%–25% [53]. Advancements in the field have allowed for the XL probe, which is currently being studied as a method to overcome adiposity interference [53]. Other factors that can limit the validity of VCTE are hepatic congestion from heart failure and cholestasis [53]. Additionally, patients must fast 3 h prior to testing [53]. Lastly, similar to most ultrasound-based imaging, VCTE is operator dependent and requires an experienced technician [53]. However, a novel numerical measurement, controlled attenuation parameter (CAP), allows for the quantification of ultrasound attenuation, correlating to the degree of steatosis; and thus, limiting operator variability [54,55].

Magnetic resonance elastography (MRE) is an imaging modality that combines magnetic resonance imaging (MRI) with mechanical waves to measure liver stiffness (Table 6) [56]. The mechanical waves are generated by a vibratory source and when they come into contact with the liver, they generate a wavelength based on liver stiffness [57]. MRI sequence then creates wave images and algorithms are then used for quantitative assessment of liver stiffness. Since Liver fibrosis does not occur as a homogenous process, especially early in its course, an advantage of MRE is that it creates a spatial map of the liver, allowing for detection of heterogenous fibrosis. Compared to VCTE, MRE is advantageous in that it has less operator dependence and is not affected by obesity [57]. Compared to the prediction models, VCTE and MRE have shown to be more accurate in predicting stage of fibrosis [58]. While the AASLD guidelines do not make any comment on preferential usage of prediction models or imaging modalities to predict fibrosis stage [11], the American Gastroenterology Association (AGA) guidelines state that there is not enough data to support use of one modality over the other [59].

### 9.2 Liver biopsy

Liver biopsy is the gold standard for assessing liver histology (Table 6) [60]. There are two main methods of obtaining a liver biopsy: percutaneous liver biopsy and transvenous liver biopsy [61]. Transvenous liver biopsy is indicated in cases of severe coagulation abnormalities, ascites, morbid obesity, atrophic liver, prior failed percutaneous biopsy, and for pressure measurements [61]. It is contraindicated to proceed with transvenous liver biopsy in the following conditions: right internal jugular vein thrombosis, hepatic vein thrombosis, hydatid cysts, and cholangitis [61]. Some of potential complications of liver biopsy include bleeding, hemoperitoneum, and fistula formation [61]. Further, there is sampling error with liver biopsy, as a liver biopsy only represents 1/50,000 of the liver parenchyma [60]. To reduce sampling error, it is recommended that at least one core biopsy be obtained and a 16-gauge needle, 2–3 cm in length, be used [11]. Further, two separate readings of a sample by one pathologist has been shown to improve diagnostic yield compared to only one reading [62].

## 10 Treatment

Treatment of NAFLD involves lifestyle modifications, treatment of comorbid medical conditions, as well as treatment of liver disease itself [11]. Patients with NAFLD should be advised to lose weight through a combination of calorie reduction and exercise, as this has been shown to reduce hepatic steatosis [11]. Additionally, NAFLD patients should be encouraged to limit heavy alcohol consumption, as more than 1.5 drinks (1 drink is defined as 12 ounces of beer, 5 ounces of wine, or 1.5 ounces of liquor) a day has been shown to increase mortality in patient with NAFLD [11,63]. Patients with comorbid diabetes and hyperlipidemia should be managed appropriately [11]. Studies have found that patients with NAFLD are not at an increased risk of hepatotoxicity from statins, and thus, statins can be used in patients with comorbid hyperlipidemia, as long as patients do not have decompensated cirrhosis [11].

While limited treatment options directly addressing liver disease are available, several studies have investigated promising therapies that target inflammation, lipid metabolism, or fibrosis (Table 7). Vitamin E and pentoxifylline are two agents that have shown to reduce inflammation in patients with NASH. The Pioglitazone versus Vitamin E versus Placebo for the Treatment of Nondiabetic Patients with NASH (PIVENS) trial showed that vitamin E significantly reduced ALT and AST levels, hepatic steatosis, and lobular inflammation [64]. It is recommended that patients with NASH without diabetes be prescribed 800 IU/day of Vitamin E for stage 2 fibrosis or higher [11,64]. Pentoxifylline is a methylxanthine derivative that reduces inflammation and may have hepatoprotective effects [65]. In a randomized placebo trial, pentoxifylline was shown to significantly reduce steatosis and lobular inflammation in patients with NASH [65].

**Table 7:** Treatment.

Therapy	Mechanism
Weight loss and Exercise	Reduce hepatic steatosis
Treat comorbid conditions (obesity, diabetes, dyslipidemia)	Reduce hepatic steatosis
Limit heavy alcohol use	Decrease inflammation
Vitamin E (antioxidant)	Decrease inflammation
Pentoxifylline (methylxanthine derivative)	Decrease inflammation
Liraglutide (glucagon-like peptide-1 analogue)	Target lipid metabolism
Obeticholic acid (farnesoid X nuclear receptor activator)	Target lipid metabolism
Elafibranor (peroxisome proliferator-activated receptor agonist)	Target lipid metabolism
Cenicriviroc (dual antagonist of C–C motif chemokine receptor types 2 and 5)	Antifibrotic

Agents currently being studied that target lipid metabolism in patients with NASH are Liraglutide (glucagon-like peptide-1 analogue), Obeticholic acid (farnesoid X nuclear receptor activator), and Elafibranor (peroxisome proliferator-activated receptor agonist) [66]. Liraglutide is commonly used for treatment in diabetes [67]. In the liraglutide safety and efficacy in patients with non-alcoholic steatohepatitis (LEAN) trial, liraglutide resulted in histological resolution of NASH [67]. Obeticholic acid leads to activation of farnesoid X nuclear receptor, leading to decreased triglyceride levels. The Farnesoid X nuclear receptor ligand obeticholic acid for non-cirrhotic NASH (FLINT) trial showed that obeticholic acid improved histological features in patients with NASH [68]. Elafibranor promotes fatty acid catabolism, which has been shown to improve dyslipidemia [66]. Currently, Elafibranor is being compared to placebo in the RESOLVE-IT phase 3 clinical study [66].

Cenicriviroc is a dual antagonist of C–C motif chemokine receptor types 2 and 5 that has been shown to have antifibrotic properties by decreasing inflammation and collagen production at the site of liver injury [66,69]. The Efficacy and Safety Study of Cenicriviroc for the Treatment of Nonalcoholic Steatohepatitis in Adult Participants with Liver Fibrosis (CENTAUR) phase 2 study showed that subjects treated with Cenicriviroc had double the improvement in fibrosis compared to placebo [69]. Although promising, larger studies are still needed for the above mentioned therapies.

## 11 Conclusion

The rise of NAFLD corresponding with the increasing prevalence of obesity, diabetes mellitus, and hypertension in the United States poses a vital opportunity for PCPs to intervene in the management and assessment of NAFLD. Although screening for NAFLD is not recommended, incidental findings on US and CT should prompt PCPs to rule out other causes of fatty liver (Table 3). Several noninvasive risk calculators, such as Fib-4, and noninvasive imaging (VCTE and MRE) can be used in the primary care setting to assess for fibrosis. Lastly, the management of NAFLD often relies on the treatment of chronic conditions mentioned earlier, through weight reduction, blood sugar control, blood pressure and cholesterol management, etc. Thus, PCPs serve as a vital subgroup of physicians at the front line of assessing and diagnosing patients with NAFLD.

## Provenance and peer review

Not commissioned, externally peer reviewed.

## Ethical approval

The paper is a review that did not involve patients; thus, ethical approval was not necessary.

## Sources of funding

The authors received no funding for this review paper.

## Author contribution

Rishi Rikhi, MD: Writing manuscript, literature review, creation of tables.

Tavankit Singh, MD: Review concept, editing manuscript, literature review.

Jamak Modaresi Esfeh, MD: Review concept, editing manuscript, literature review, creation of figures.

## Research registration

The paper is a review that did not involve human subjects; thus, a registry was not necessary.

## Guarantor

The paper was created and written by all, and only, the authors listed: Rishi Rikhi, MD, Tavankit Singh, MD, and Jamak Modaresi Esfeh, MD. There were no other members who had access to the study, data, or controlled the decision to publish the work.

## Consent

The paper is a review that did not involve patients or volunteers; thus, ethical approval and informed written consent was not necessary.

## Declaration of competing interest

The authors have no conflicts of interest.

## References

[1] Murphy S.L., Xu J., Kochanek K.D., Curtin S.C., Arias E. (2017). Deaths: final data for 2015. Natl. Vital Stat. Rep..

[2] Perumpail B.J., Khan M.A., Yoo E.R., Cholankeril G., Kim D., Ahmed A. (2017). Clinical epidemiology and disease burden of nonalcoholic fatty liver disease. World J. Gastroenterol..

[3] Younossi Z.M., Koenig A.B., Abdelatif D., Fazel Y., Henry L., Wymer M. (2016). Global epidemiology of nonalcoholic fatty liver disease-Meta-analytic assessment of prevalence, incidence, and outcomes. Hepatology.

[4] Younossi Z.M., Stepanova M., Afendy M., Fang Y., Younossi Y., Mir H., Srishord M. (2011). Changes in the prevalence of the most common causes of chronic liver diseases in the United States from 1988 to 2008. Clin. Gastroenterol. Hepatol..

[5] Kim W.R., Lake J.R., Smith J.M., Schladt D.P., Skeans M.A., Harper A.M., Wainright J.L., Snyder J.J., Israni A.K., Kasiske B.L. (2018). OPTN/SRTR 2016 annual data report: liver. Am. J. Transplant..

[6] Canbay A., Sowa J.P., Syn W.K., Treckmann J. (2016). NASH cirrhosis - the new burden in liver transplantation: how should it Be managed?. Vis. Med..

[7] Ofosu A., Ramai D., Reddy M. (2018). Non-alcoholic fatty liver disease: controlling an emerging epidemic, challenges, and future directions. Ann. Gastroenterol..

[8] Tomizawa M., Kawanabe Y., Shinozaki F., Sato S., Motoyoshi Y., Sugiyama T., Yamamoto S., Sueishi M. (2014). Triglyceride is strongly associated with nonalcoholic fatty liver disease among markers of hyperlipidemia and diabetes. Biomed. Rep..

[9] Wieland A.C., Quallick M., Truesdale A., Mettler P., Bambha K.M. (2013). Identifying practice gaps to optimize medical care for patients with nonalcoholic fatty liver disease. Dig. Dis. Sci..

[11] Chalasani N., Younossi Z., Lavine J.E., Charlton M., Cusi K., Rinella M., Harrison S.A., Brunt E.M., Sanyal A.J. (2018). The diagnosis and management of nonalcoholic fatty liver disease: practice guidance from the American Association for the Study of Liver Diseases. Hepatology.

[12] Savolainen V.T., Liesto K., Mannikko A., Penttila A., Karhunen P.J. (1993). Alcohol consumption and alcoholic liver disease: evidence of a threshold level of effects of ethanol. Alcohol Clin. Exp. Res..

[13] Hassan K., Bhalla V., El Regal M.E., HH A.K. (2014). Nonalcoholic fatty liver disease: a comprehensive review of a growing epidemic. World J. Gastroenterol..

[14] Singh S., Allen A.M., Wang Z., Prokop L.J., Murad M.H., Loomba R. (2015). Fibrosis progression in nonalcoholic fatty liver vs nonalcoholic steatohepatitis: a systematic review and meta-analysis of paired-biopsy studies. Clin. Gastroenterol. Hepatol..

[15] Spengler E.K., Loomba R. (2015). Recommendations for diagnosis, referral for liver biopsy, and treatment of nonalcoholic fatty liver disease and nonalcoholic steatohepatitis. Mayo Clin. Proc..

[16] Bedossa P. (2017). Pathology of non-alcoholic fatty liver disease. Liver Int..

[17] Kleiner D.E., Brunt E.M., Van Natta M., Behling C., Contos M.J., Cummings O.W., Ferrell L.D., Liu Y.C., Torbenson M.S., Unalp-Arida A., Yeh M., McCullough A.J., Sanyal A.J., Nonalcoholic Steatohepatitis Clinical Research N. (2005). Design and validation of a histological scoring system for nonalcoholic fatty liver disease. Hepatology.

[18] Matteoni C.A., Younossi Z.M., Gramlich T., Boparai N., Liu Y.C., McCullough A.J. (1999). Nonalcoholic fatty liver disease: a spectrum of clinical and pathological severity. Gastroenterology.

[19] Yki-Jarvinen H. (2014). Non-alcoholic fatty liver disease as a cause and a consequence of metabolic syndrome. Lancet Diabetes Endocrinol..

[20] Muse E.D., Obici S., Bhanot S., Monia B.P., McKay R.A., Rajala M.W., Scherer P.E., Rossetti L. (2004). Role of resistin in diet-induced hepatic insulin resistance. J. Clin. Investig..

[21] Xu A., Wang Y., Keshaw H., Xu L.Y., Lam K.S., Cooper G.J. (2003). The fat-derived hormone adiponectin alleviates alcoholic and nonalcoholic fatty liver diseases in mice. J. Clin. Investig..

[22] Buzzetti E., Pinzani M., Tsochatzis E.A. (2016). The multiple-hit pathogenesis of non-alcoholic fatty liver disease (NAFLD). Metabolism.

[23] Lonardo A., Nascimbeni F., Mantovani A., Targher G. (2018). Hypertension, diabetes, atherosclerosis and NASH: cause or consequence?. J. Hepatol..

[24] Kaur J. (2014). A comprehensive review on metabolic syndrome. Cardiol. Res. Pract..

[25] Bellentani S., Scaglioni F., Marino M., Bedogni G. (2010). Epidemiology of non-alcoholic fatty liver disease. Dig. Dis..

[26] Lonardo A., Nascimbeni F., Maurantonio M., Marrazzo A., Rinaldi L., Adinolfi L.E. (2017). Nonalcoholic fatty liver disease: evolving paradigms. World J. Gastroenterol..

[27] Saab S., Manne V., Nieto J., Schwimmer J.B., Chalasani N.P. (2016). Nonalcoholic fatty liver disease in latinos. Clin. Gastroenterol. Hepatol..

[28] Setiawan V.W., Stram D.O., Porcel J., Lu S.C., Le Marchand L., Noureddin M. (2016). Prevalence of chronic liver disease and cirrhosis by underlying cause in understudied ethnic groups: the multiethnic cohort. Hepatology.

[29] Williams C.D., Stengel J., Asike M.I., Torres D.M., Shaw J., Contreras M., Landt C.L., Harrison S.A. (2011). Prevalence of nonalcoholic fatty liver disease and nonalcoholic steatohepatitis among a largely middle-aged population utilizing ultrasound and liver biopsy: a prospective study. Gastroenterology.

[30] Kneeman J.M., Misdraji J., Corey K.E. (2012). Secondary causes of nonalcoholic fatty liver disease. Ther. Adv. Gastroenterol..

[31] Mofrad P., Contos M.J., Haque M., Sargeant C., Fisher R.A., Luketic V.A., Sterling R.K., Shiffman M.L., Stravitz R.T., Sanyal A.J. (2003). Clinical and histologic spectrum of nonalcoholic fatty liver disease associated with normal ALT values. Hepatology.

[32] Sanyal D., Mukherjee P., Raychaudhuri M., Ghosh S., Mukherjee S., Chowdhury S. (2015). Profile of liver enzymes in non-alcoholic fatty liver disease in patients with impaired glucose tolerance and newly detected untreated type 2 diabetes. Indian J. Endocrinol. Metab..

[33] Gowda S., Desai P.B., Hull V.V., Math A.A., Vernekar S.N., Kulkarni S.S. (2009). A review on laboratory liver function tests. Pan Afr. Med. J..

[34] Giannini E.G., Testa R., Savarino V. (2005). Liver enzyme alteration: a guide for clinicians. CMAJ (Can. Med. Assoc. J.).

[35] Lee D.H. (2017). Imaging evaluation of non-alcoholic fatty liver disease: focused on quantification. Clin. Mol. Hepatol..

[36] Hamer O.W., Aguirre D.A., Casola G., Lavine J.E., Woenckhaus M., Sirlin C.B. (2006). Fatty liver: imaging patterns and pitfalls. RadioGraphics.

[37] Dasarathy S., Dasarathy J., Khiyami A., Joseph R., Lopez R., McCullough A.J. (2009). Validity of real time ultrasound in the diagnosis of hepatic steatosis: a prospective study. J. Hepatol..

[38] Tapper E.B., Rahni D.O., Arnaout R., Lai M. (2013). The overuse of serum ceruloplasmin measurement. Am. J. Med..

[39] Corey K.E., Klebanoff M.J., Tramontano A.C., Chung R.T., Hur C. (2016). Screening for nonalcoholic steatohepatitis in individuals with type 2 diabetes: a cost-effectiveness analysis. Dig. Dis. Sci..

[40] Wong V.W., Chalasani N. (2016). Not routine screening, but vigilance for chronic liver disease in patients with type 2 diabetes. J. Hepatol..

[41] Angulo P., Kleiner D.E., Dam-Larsen S., Adams L.A., Bjornsson E.S., Charatcharoenwitthaya P., Mills P.R., Keach J.C., Lafferty H.D., Stahler A., Haflidadottir S., Bendtsen F. (2015). Liver fibrosis, but No other histologic features, is associated with long-term outcomes of patients with nonalcoholic fatty liver disease. Gastroenterology.

[42] Cheah M.C., McCullough A.J., Goh G.B. (2017). Current modalities of fibrosis assessment in non-alcoholic fatty liver disease. J. Clin. Transl. Hepatol..

[43] Angulo P., Hui J.M., Marchesini G., Bugianesi E., George J., Farrell G.C., Enders F., Saksena S., Burt A.D., Bida J.P., Lindor K., Sanderson S.O., Lenzi M., Adams L.A., Kench J., Therneau T.M., Day C.P. (2007). The NAFLD fibrosis score: a noninvasive system that identifies liver fibrosis in patients with NAFLD. Hepatology.

[44] Tapper E.B., Hunink M.G., Afdhal N.H., Lai M., Sengupta N. (2016). Cost-effectiveness analysis: risk stratification of nonalcoholic fatty liver disease (NAFLD) by the primary care physician using the NAFLD fibrosis score. PLoS One.

[45] Sterling R.K., Lissen E., Clumeck N., Sola R., Correa M.C., Montaner J., Sulkowski M.S., Torriani F.J., Dieterich D.T., Thomas D.L., Messinger D., Nelson M., Investigators A.C. (2006). Development of a simple noninvasive index to predict significant fibrosis in patients with HIV/HCV coinfection. Hepatology.

[46] Shah A.G., Lydecker A., Murray K., Tetri B.N., Contos M.J., Sanyal A.J., Nash Clinical Research N. (2009). Comparison of noninvasive markers of fibrosis in patients with nonalcoholic fatty liver disease. Clin. Gastroenterol. Hepatol..

[47] Kim B.K., Kim D.Y., Park J.Y., Ahn S.H., Chon C.Y., Kim J.K., Paik Y.H., Lee K.S., Park Y.N., Han K.H. (2010). Validation of FIB-4 and comparison with other simple noninvasive indices for predicting liver fibrosis and cirrhosis in hepatitis B virus-infected patients. Liver Int..

[48] McPherson S., Stewart S.F., Henderson E., Burt A.D., Day C.P. (2010). Simple non-invasive fibrosis scoring systems can reliably exclude advanced fibrosis in patients with non-alcoholic fatty liver disease. Gut.

[49] Schachter J.L., Patel M., Horton S.R., Mike Devane A., Ewing A., Abrams G.A. (2018). FibroSURE and elastography poorly predict the severity of liver fibrosis in Fontan-associated liver disease. Congenit. Heart Dis..

[50] Huang Y., Adams L.A., Joseph J., Bulsara M.K., Jeffrey G.P. (2017). The ability of Hepascore to predict liver fibrosis in chronic liver disease: a meta-analysis. Liver Int..

[51] Xie Q., Zhou X., Huang P., Wei J., Wang W., Zheng S. (2014). The performance of enhanced liver fibrosis (ELF) test for the staging of liver fibrosis: a meta-analysis. PLoS One.

[52] Vuppalanchi R., Siddiqui M.S., Van Natta M.L., Hallinan E., Brandman D., Kowdley K., Neuschwander-Tetri B.A., Loomba R., Dasarathy S., Abdelmalek M., Doo E., Tonascia J.A., Kleiner D.E., Sanyal A.J., Chalasani N., Network N.C.R. (2018). Performance characteristics of vibration-controlled transient elastography for evaluation of nonalcoholic fatty liver disease. Hepatology.

[53] Tapper E.B., Castera L., Afdhal N.H. (2015). FibroScan (vibration-controlled transient elastography): where does it stand in the United States practice. Clin. Gastroenterol. Hepatol..

[54] Karlas T., Petroff D., Sasso M., Fan J.G., Mi Y.Q., de Ledinghen V., Kumar M., Lupsor-Platon M., Han K.H., Cardoso A.C., Ferraioli G., Chan W.K., Wong V.W., Myers R.P., Chayama K., Friedrich-Rust M., Beaugrand M., Shen F., Hiriart J.B., Sarin S.K., Badea R., Jung K.S., Marcellin P., Filice C., Mahadeva S., Wong G.L., Crotty P., Masaki K., Bojunga J., Bedossa P., Keim V., Wiegand J. (2017). Individual patient data meta-analysis of controlled attenuation parameter (CAP) technology for assessing steatosis. J. Hepatol..

[55] Sasso M., Beaugrand M., de Ledinghen V., Douvin C., Marcellin P., Poupon R., Sandrin L., Miette V. (2010). Controlled attenuation parameter (CAP): a novel VCTE guided ultrasonic attenuation measurement for the evaluation of hepatic steatosis: preliminary study and validation in a cohort of patients with chronic liver disease from various causes. Ultrasound Med. Biol..

[56] Dulai P.S., Sirlin C.B., Loomba R. (2016). MRI and MRE for non-invasive quantitative assessment of hepatic steatosis and fibrosis in NAFLD and NASH: clinical trials to clinical practice. J. Hepatol..

[57] Venkatesh S.K., Yin M., Ehman R.L. (2013). Magnetic resonance elastography of liver: technique, analysis, and clinical applications. J. Magn. Reson. Imaging.

[58] Xiao G., Zhu S., Xiao X., Yan L., Yang J., Wu G. (2017). Comparison of laboratory tests, ultrasound, or magnetic resonance elastography to detect fibrosis in patients with nonalcoholic fatty liver disease: a meta-analysis. Hepatology.

[59] Lim J.K., Flamm S.L., Singh S., Falck-Ytter Y.T., Clinical A. (2017). Guidelines committee of the American gastroenterological, American gastroenterological association institute guideline on the role of elastography in the evaluation of liver fibrosis. Gastroenterology.

[60] Nalbantoglu I.L., Brunt E.M. (2014). Role of liver biopsy in nonalcoholic fatty liver disease. World J. Gastroenterol..

[61] Dohan A., Guerrache Y., Boudiaf M., Gavini J.P., Kaci R., Soyer P. (2014). Transjugular liver biopsy: indications, technique and results. Diagn. Interventional Imag..

[62] Vuppalanchi R., Unalp A., Van Natta M.L., Cummings O.W., Sandrasegaran K.E., Hameed T., Tonascia J., Chalasani N. (2009). Effects of liver biopsy sample length and number of readings on sampling variability in nonalcoholic Fatty liver disease. Clin. Gastroenterol. Hepatol..

[63] Hajifathalian K., Torabi Sagvand B., McCullough A.J. (2018). Effect of alcohol consumption on survival in nonalcoholic fatty liver disease: a national prospective cohort study. Hepatology.

[64] Sanyal A.J., Chalasani N., Kowdley K.V., McCullough A., Diehl A.M., Bass N.M., Neuschwander-Tetri B.A., Lavine J.E., Tonascia J., Unalp A., Van Natta M., Clark J., Brunt E.M., Kleiner D.E., Hoofnagle J.H., Robuck P.R., Nash C.R.N., Pioglitazone, vitamin E. (2010). Or placebo for nonalcoholic steatohepatitis. N. Engl. J. Med..

[65] Zein C.O., Yerian L.M., Gogate P., Lopez R., Kirwan J.P., Feldstein A.E., McCullough A.J. (2011). Pentoxifylline improves nonalcoholic steatohepatitis: a randomized placebo-controlled trial. Hepatology.

[66] Connolly J.J., Ooka K., Lim J.K. (2018). Future pharmacotherapy for non-alcoholic steatohepatitis (NASH): review of phase 2 and 3 trials. J. Clin. Transl. Hepatol..

[67] Armstrong M.J., Gaunt P., Aithal G.P., Barton D., Hull D., Parker R., Hazlehurst J.M., Guo K., team L.t., Abouda G., Aldersley M.A., Stocken D., Gough S.C., Tomlinson J.W., Brown R.M., Hubscher S.G., Newsome P.N. (2016). Liraglutide safety and efficacy in patients with non-alcoholic steatohepatitis (LEAN): a multicentre, double-blind, randomised, placebo-controlled phase 2 study. Lancet.

[68] Neuschwander-Tetri B.A., Loomba R., Sanyal A.J., Lavine J.E., Van Natta M.L., Abdelmalek M.F., Chalasani N., Dasarathy S., Diehl A.M., Hameed B., Kowdley K.V., McCullough A., Terrault N., Clark J.M., Tonascia J., Brunt E.M., Kleiner D.E., Doo E., Network N.C.R. (2015). Farnesoid X nuclear receptor ligand obeticholic acid for non-cirrhotic, non-alcoholic steatohepatitis (FLINT): a multicentre, randomised, placebo-controlled trial. Lancet.

[69] Friedman S.L., Ratziu V., Harrison S.A., Abdelmalek M.F., Aithal G.P., Caballeria J., Francque S., Farrell G., Kowdley K.V., Craxi A., Simon K., Fischer L., Melchor-Khan L., Vest J., Wiens B.L., Vig P., Seyedkazemi S., Goodman Z., Wong V.W., Loomba R., Tacke F., Sanyal A., Lefebvre E. (2018). A randomized, placebo-controlled trial of cenicriviroc for treatment of nonalcoholic steatohepatitis with fibrosis. Hepatology.

